# Systematic analysis of echinocandin-associated adverse reactions using the FAERS database: identification of unique DRESS and resistance signals

**DOI:** 10.3389/fmed.2026.1957674

**Published:** 2026-09-28

**Authors:** Xue Zhang, Ji Nie, Yuanchun Xie, Ya Ran, Hanhan Zhou, Bin Li

**Affiliations:** Department of Pharmacy, The People's Hospital of Yubei District of Chongqing City, Chongqing, China

**Keywords:** adverse drug reactions, caspofungin, echinocandins, FAERS, pharmacovigilance

## Abstract

**Aim:**

This pharmacovigilance study analyzed echinocandin-related adverse events (AEs) using the FDA Adverse Event Reporting System (FAERS) database to identify agent-specific safety signals and inform clinical decision-making.

**Methods:**

AE reports were extracted from FAERS covering Q1 2004 to Q4 2025. Four disproportionality algorithms—reporting odds ratio (ROR), proportional reporting ratio (PRR), information component (IC025), and empirical Bayesian geometric mean (EBGM05)—were applied. Positive signals were defined when all four methods yielded positive results.

**Results:**

A total of 5,298 cases with echinocandins as the primary suspect drug were included. Caspofungin showed unique association with DRESS syndrome (ROR = 16.62, IC025 = 3.21). Exceptionally strong resistance signals were detected for caspofungin (“pathogen resistance”: ROR = 68.83; “drug resistance”: ROR = 22.32) and micafungin (“bronchopulmonary aspergillosis”: ROR = 71.99; “Candida infection”: ROR = 23.93). Hepatobiliary signals included cholestasis (ROR = 24.97) and alkaline phosphatase increase (ROR = 19.10) for caspofungin, with similar signals observed for micafungin and anidulafungin.

**Conclusion:**

This pharmacovigilance study identified two clinically significant signals: DRESS as a unique safety concern for caspofungin, and striking resistance signals for caspofungin and micafungin. These findings emphasize the need for vigilant monitoring during echinocandin therapy and reinforce antifungal stewardship.

## 1 Introduction

The increasing prevalence and emerging drug resistance of pathogenic fungi present a significant threat to human health (1). Echinocandins (caspofungin, micafungin, anidulafungin, and rezafungin) are a novel class of semisynthetic cyclic lipopeptide antifungal agents. By noncompetitively inhibiting the *β*-(1,3)-D-glucan synthase complex located in the fungal cell wall, echinocandins disrupt cell wall integrity, leading to osmotic lysis and subsequent fungicidal activity (2). Their mechanism of action targets a structure absent in mammalian cells, conferring a favorable therapeutic index with minimal off-target toxicity (3). They have demonstrated high efficacy in the treatment of invasive fungal infections and against fungal biofilms, and are recommended as first-line therapy for invasive candidiasis as well as an alternative treatment for invasive aspergillosis (4, 5). In addition, they are widely used for empirical antifungal therapy in neutropenic patients and as salvage therapy for refractory invasive fungal infections (6).

The global incidence of invasive fungal infections (IFIs) has risen substantially over recent decades, driven by an expanding immunocompromised population-including recipients of hematopoietic stem cell transplantation (HSCT), solid organ transplantation (SOT), patients with hematological malignancies, and those receiving intensive immunosuppressive therapies (7). Consequently, echinocandin prescriptions have increased correspondingly. Although regulatory trials and early post-marketing studies have generally confirmed the safety and tolerability of echinocandins, accumulating case reports and observational studies have raised concerns about specific adverse events (8, 9). These include hepatotoxicity (elevated transaminases, cholestasis), infusion-related reactions (flushing, rash, bronchospasm), hematological abnormalities (eosinophilia, thrombocytopenia), and rare but severe adverse events such as complete heart block (10–12).

Moreover, the emergence of echinocandin resistance—primarily mediated by FKS gene hotspot mutations—has become an increasingly recognized clinical challenge, particularly among *Candida glabrata* (*Nakaseomyces glabratus*) and *Candida parapsilosis* isolates (13, 14). Treatment failure due to acquired or intrinsic resistance may manifest as persistent fungemia, breakthrough infections, or mycological relapse, which may be captured in pharmacovigilance databases as AEs such as “drug ineffective,” “pathogen resistance,” or “fungal infection.” Therefore, systematic real-world assessment of effectiveness and safety signals is essential for guiding clinical practice.

Spontaneous reporting systems, notably the FDA Adverse Event Reporting System (FAERS), have unique advantages for post-marketing safety surveillance. FAERS contains over 20 million AE reports submitted by healthcare professionals, consumers, and manufacturers from more than 100 countries (15). Data mining of FAERS using disproportionality analyses enables the detection of potential drug–AE associations, especially for rare, delayed, or serious events that may be missed in pre-marketing trials. Prior pharmacovigilance studies have investigated individual echinocandins (16), but systematic comparisons across all four agents using standardized statistical thresholds are lacking. Furthermore, the recent approval and increasing use of rezafungin (first approved by FDA in 2023 for candidemia and invasive candidiasis) necessitate updated real-world evidence.

To address these gaps, we performed a comprehensive retrospective analysis of echinocandin-associated AEs using FAERS data from the first quarter (Q1) 2004 to the fourth quarter (Q4) 2025. We applied four complementary disproportionality methods, Reporting Odds Ratio (ROR), Proportional Reporting Ratio (PRR), Information Component (IC025), Empirical Bayesian Geometric Mean (EBGM) to: characterize demographic and clinical features of echinocandin-related AEs; describe temporal trends and time-to-onset distributions; identify positive safety signals at both PT and SOC levels; and compare signals across individual agents where data permit. Our findings aim to provide evidence-based insights for optimizing the benefit-risk assessment of echinocandins in clinical practice.

## 2 Materials and methods

### 2.1 Data source

We extracted data from the FAERS, a publicly available database that collects voluntary AE reports from healthcare professionals (physicians, pharmacists, nurses), consumers, lawyers, and pharmaceutical manufacturers. The database includes information on patient demographics (age, sex, weight), reporter qualifications, report countries, suspect drugs (including role codes: primary suspect, secondary suspect, concomitant, or interacting), drug indications, All AE terms were coded using the Medical Dictionary for Regulatory Activities (MedDRA, version 27.0); reports from earlier periods were recoded to this version to ensure consistent classification across the full study window, outcomes (death, life-threatening, hospitalization, disability, congenital anomaly, required intervention, other serious), and report dates.

The study period spanned from Q1 2004 to Q4 2025. We included four echinocandin active ingredients: caspofungin, micafungin, anidulafungin, and rezafungin. Both brand and generic names were considered. Data were downloaded as quarterly ASCII files and imported into R for processing.

### 2.2 Data cleaning and deduplication

We followed the FDA-recommended deduplication procedure: for reports sharing the same CASEID, we retained the record with the latest FDA_DT; if CASEID and FDA_DT were identical, we kept the record with the highest PRIMARYID. For descriptive demographic analyses, reports with implausible age (<0 or >120 years) or sex (not “Male” or “Female”) were excluded; weight data were summarized as reported in the FAERS database, with the observed range being 0.30–250.00 kg. For disproportionality analyses, cases with missing demographic data were retained to avoid selection bias, as missingness is common in spontaneous reporting systems. Only records where an echinocandin was coded as the primary suspect (PS) drug were included to reduce confounding from concomitant medications. Reports where the primary PT was specifically related to procedural complications (e.g., procedural site reactions, postoperative complications) were removed. However, the SOC “Injury, poisoning and procedural complications” also contains non-procedural PTs (e.g., “Off label use,” “Product prescribing issue”), which were retained. Reports involving combination antifungal therapy (e.g., echinocandin plus voriconazole) were excluded to minimize false-positive signals due to drug interactions. Two independent researchers performed data processing and validation; discrepancies were resolved by a third researcher.

### 2.3 Statistical analysis

We conducted disproportionality analysis using four standard algorithms: ROR: Ratio of the odds of a specific AE in the target drug group versus all other drugs in the database. A positive signal was defined as ROR with lower limit of 95% confidence interval (ROR025) > 1, with at least three cases. PRR: Ratio of the proportion of a specific AE for the target drug versus all other drugs. PRR ≥ 2, *χ*^2^ ≥ 4, and case count ≥ 3 indicated a signal. IC: A Bayesian measure where the observed-to-expected ratio is logarithmically transformed. IC025 (lower limit of 95% confidence interval) > 0 was considered significant. EBGM: A shrinkage estimator that reduces false positives for rare events. EBGM05 (lower bound of 95% confidence interval) > 2 was considered a signal. An AE was classified as a positive signal only if all four methods simultaneously yielded positive results. We also summarized the demographic characteristics, report sources, geographical distribution, and serious outcomes using descriptive statistics. Time-to-onset (TTO) was calculated as the difference between the AE onset date and the drug start date; only positive values were included. Median TTO and interquartile ranges (IQR) were calculated using reports with complete date information. The SOC-level distribution was presented as a horizontal bar chart showing the percentage of reports per SOC for each drug. The denominator for each drug was the total number of reports for that drug. For reports with multiple PTs, each PT was counted independently and assigned to its corresponding SOC; no report-level deduplication was applied at the SOC level.

## 3 Results

### 3.1 Study population characteristics

From Q1 2004 to Q4 2025, a total of 5,298 cases with echinocandins as the primary suspect (PS) drug were included after data cleaning and deduplication. The analysis covered four active ingredients: caspofungin (2,408 cases, 45.5%), micafungin (2,375 cases, 44.8%), anidulafungin (416 cases, 7.9%), and rezafungin (99 cases, 1.9%). Among cases with documented sex, males accounted for 2,774 (59.6%) and females for 1,877 (40.4%); both caspofungin and micafungin exhibited male proportions exceeding 52% of their respective drug-specific totals. The age distribution was predominantly middle-aged and elderly: 45–64 years (1,330 cases, 26.9%) and ≥65 years (1,287 cases, 24.3%); patients aged <18 years comprised 465 cases (9.4%). Weight data were available for 1,304 cases, with an overall mean of 59.98 kg (SD = 28.35). Physicians were the most frequent reporters (2,145 cases, 40.5%), followed by consumers (1,232 cases, 23.3%) and pharmacists (1,024 cases, 19.3%). The United States contributed the highest number of reports (1,407 cases, 26.6%), followed by Japan (837 cases, 15.8%), France (551 cases, 10.4%), and China (445 cases, 8.4%). Regarding serious outcomes, death was the most frequently reported (1,868 cases, 35.3%), followed by hospitalization (initial or prolonged) (1,336 cases, 25.2%) and life-threatening events (477 cases, 9.0%). Detailed demographic and clinical characteristics are presented in Table 1.

**Table 1:** Basic characteristics of echinocandins in FAERS.

Characteristic	Caspofungin (%)	Micafungin (%)	Anidulafungin (%)	Rezafungin (%)	Total
Total cases	2408	2375	416	99	5298
Gender					
Female	862 (35.80)	840 (35.37)	137 (32.93)	38 (38.38)	1877 (35.43)
Male	1255 (52.12)	1289 (54.27)	199 (47.84)	31 (31.31)	2774 (52.36)
Not specified	291 (12.08)	246 (10.36)	80 (19.23)	30 (30.30)	647 (12.21)
Age (years)					
<18	268 (11.13)	177 (7.45)	19 (4.57)	1 (1.01)	465 (9.42)
18–44	414 (17.19)	344 (14.48)	63 (15.14)	9 (9.09)	830 (16.81)
45–64	674 (27.99)	556 (23.41)	91 (21.88)	9 (9.09)	1330 (26.93)
≥65	591 (24.54)	565 (23.79)	117 (28.13)	14 (14.14)	1287 (24.29)
Not specified	461 (19.14)	733 (30.86)	126 (30.29)	66 (66.67)	1386 (26.16)
Weight (kg)					
N (missing)	686 (1722)	500 (1875)	106 (310)	12 (87)	1304 (3994)
Mean (SD)	62.34 (25.97)	54.33 (30.85)	70.73 (26.58)	65.81 (17.39)	59.98 (28.35)
Median (Q1,Q3)	60.00 (48.00,75.00)	52.05 (40.00,67.29)	70.00 (58.00,83.00)	65.80 (54.50,80.50)	59.00 (46.00,73.00)
Min, Max	0.75, 250.00	0.30, 240.00	2.46, 164.00	32.00, 95.00	0.30,250.00
Reporters					
Consumer	228 (9.47)	985 (41.47)	17 (4.09)	2 (2.02)	1232 (23.25)
Not specified	66 (2.74)	46 (1.93)	34 (8.17)	22 (22.22)	168 (3.17)
Other health-professional	391 (16.24)	293 (12.34)	45 (10.82)	0 (0)	729 (13.76)
Pharmacist	593 (24.63)	307 (12.93)	105 (25.24)	19 (19.19)	1024 (19.33)
Physician	1130 (46.93)	744 (31.33)	215 (51.68)	56 (56.57)	2145 (40.49)
Report countries					
USA	386 (16.03)	880 (37.05)	73 (17.55)	68 (68.69)	1407 (26.56)
France	401 (16.65)	144 (6.06)	3 (0.72)	0 (0.00)	551 (10.40)
Japan	298 (12.38)	539 (22.69)	0 (0.00)	0 (0.00)	837 (15.80)
United Kingdom	80 (3.32)	44 (1.85)	26 (6.25)	4 (4.04)	154 (2.91)
China	141 (5.86)	304 (12.80)	0 (0.00)	0 (0.00)	445 (8.40)
Other countries	1243 (51.62)	464 (19.54)	314 (75.48)	27 (27.27)	1904 (35.94)
Outcome					
Life-threatening	257 (10.67)	164 (6.91)	52 (12.50)	4 (4.04)	477 (9.00)
Hospitalization - initial or prolonged	791 (32.85)	436 (18.36)	94 (22.60)	15 (15.15)	1336 (25.22)
Disability	71 (2.95)	33 (1.39)	3 (0.72)	0 (0.00)	107 (2.02)
Death	943 (39.16)	763 (32.13)	147 (35.34)	15 (15.15)	1868 (35.26)
Congenital anomaly	1 (0.04)	2 (0.08)	0 (0.00)	0 (0.00)	3 (0.06)
Required intervention to prevent permanent impairment/damage	7 (0.29)	5 (0.21)	1 (0.24)	1 (1.01)	14 (0.26)
Other	1074 (44.60)	1066 (44.88)	242 (58.17)	32 (32.32)	2414 (45.56)

### 3.2 Annual trends in adverse event reports

As illustrated in Figure 1, the annual number of adverse event (AE) reports for the four echinocandins exhibited distinct temporal trends from 2004 to 2025, reflecting differences in approval dates, extent of clinical use, and the progressive development of the FAERS database.

**Figure 1:**
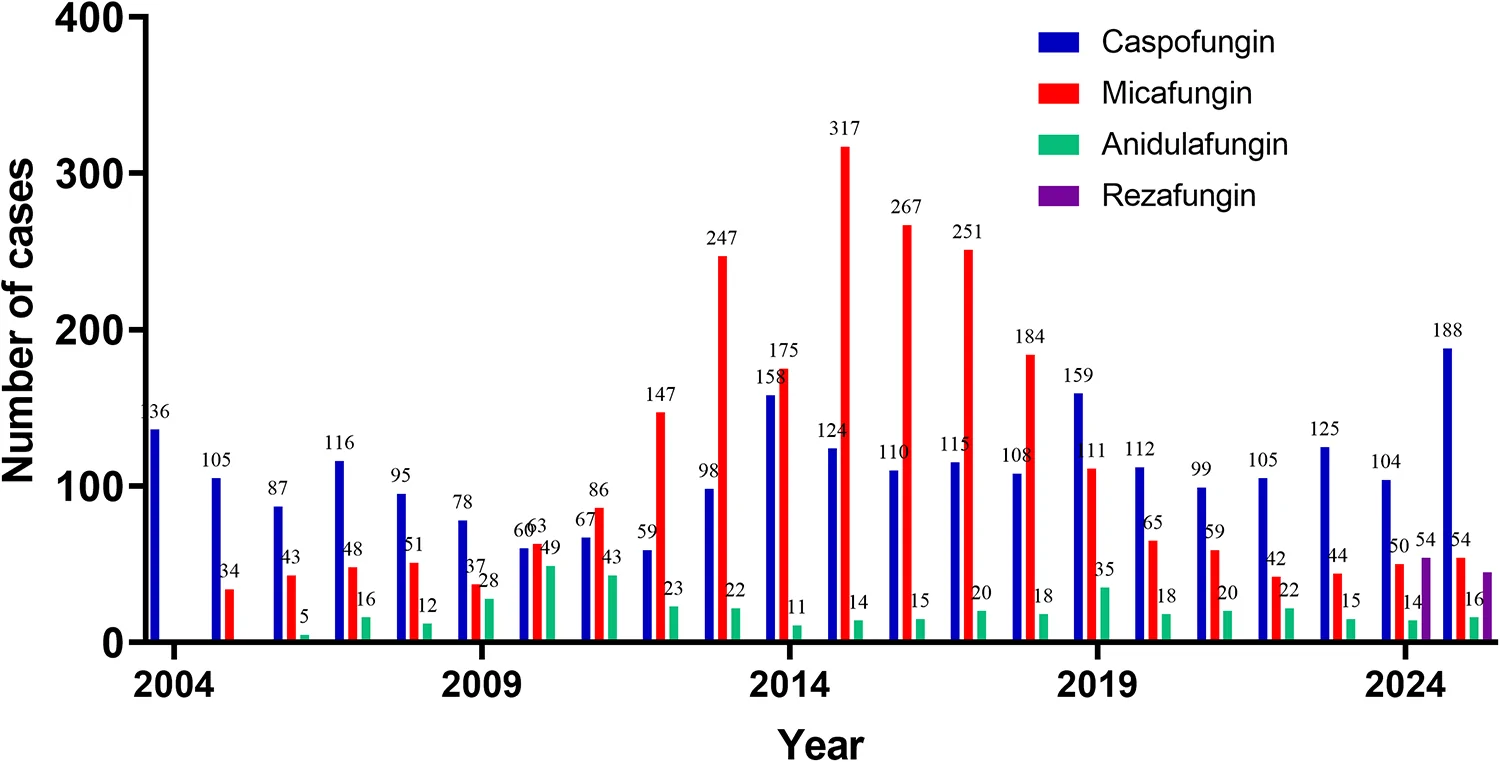
Echinocandins AE reports (2004−2025). The *x*-axis shows the reporting year, and the *y*-axis displays the number of reports, illustrating the yearly volume of AE submissions related to echinocandins.

As the first approved echinocandin, caspofungin received FDA approval on January 26, 2001 (brand name Cancidas). AE reports in the FAERS database begin in 2004, which reflects the start of publicly available quarterly data files rather than the true onset of reporting. AE reports totaled 136 cases in 2004, decreased to 105 in 2005 and 87 in 2006, then fluctuated upward. Peaks were observed in 2014 (158 cases) and 2019 (159 cases), with 2025 reporting 188 cases-the highest annual figure to date—reflecting a generally increasing trend.

Micafungin was approved by the FDA on March 16, 2005. Reports first appeared in 2005 with 34 cases and increased steadily thereafter, peaking at 317 cases in 2015, followed by 267 (2016), 251 (2017), and 184 (2018), before gradually declining to 54 cases in 2025. This drug had the highest historical reporting volume among the four echinocandins.

Anidulafungin was approved by the FDA on February 17, 2006. First reported in 2006 (5 cases), rose to 49 cases in 2010 and 43 in 2011, but most years remained below 30 cases. 2025 recorded 16 cases. Overall, anidulafungin had the lowest report counts throughout the study period.

As the newest echinocandin, rezafungin received FDA approval on March 22, 2023 (brand name Rezzayo). The database first captured its AE reports in 2024 (54 cases), followed by 45 cases in 2025. Long-term trends cannot be established due to its recent approval, but the rapid reporting volume in the early post-market period warrants continued pharmacovigilance.

Micafungin had the highest historical reporting volume, peaking in 2015 (317 cases). Caspofungin showed a fluctuating upward trend, reaching 188 cases in 2025. Anidulafungin consistently had low report counts. Rezafungin, the most recently approved echinocandin, first appeared in 2024 and requires ongoing safety monitoring.

### 3.3 Time-to-onset distribution of adverse events

The AE reporting pattern associated with Echinocandins exhibits a distinct time-dependent trend. As shown in Figure 2, we analyzed the time interval from drug initiation to AE onset. Among the 5,298 included reports, 3,185 (60.1%) lacked complete date information and were excluded from the TTO analysis—a well-recognized limitation of spontaneous reporting systems such as FAERS. The remaining 2,113 reports with documented dates were distributed across time-to-onset intervals. For caspofungin, 909 reports had complete dates; for micafungin, 1,033; for anidulafungin, 158; and for rezafungin, 13. The median TTO was 4 days (IQR: 1–11) for caspofungin, 2 days (IQR: 0–8) for micafungin, 1 day (IQR: 0–10) for anidulafungin, and 2 days (IQR: 0–19) for rezafungin. Notably, rezafungin had only 13 reports with complete dates, reflecting its recent market approval (2023). From a clinical perspective, echinocandins are primarily administered to hospitalized patients with invasive fungal infections; thus, AEs might be more concentrated in the early treatment phase (0–30 days). Nevertheless, the current data do not permit a precise time-to-event analysis, and validation using original individual-level data is warranted.

**Figure 2:**
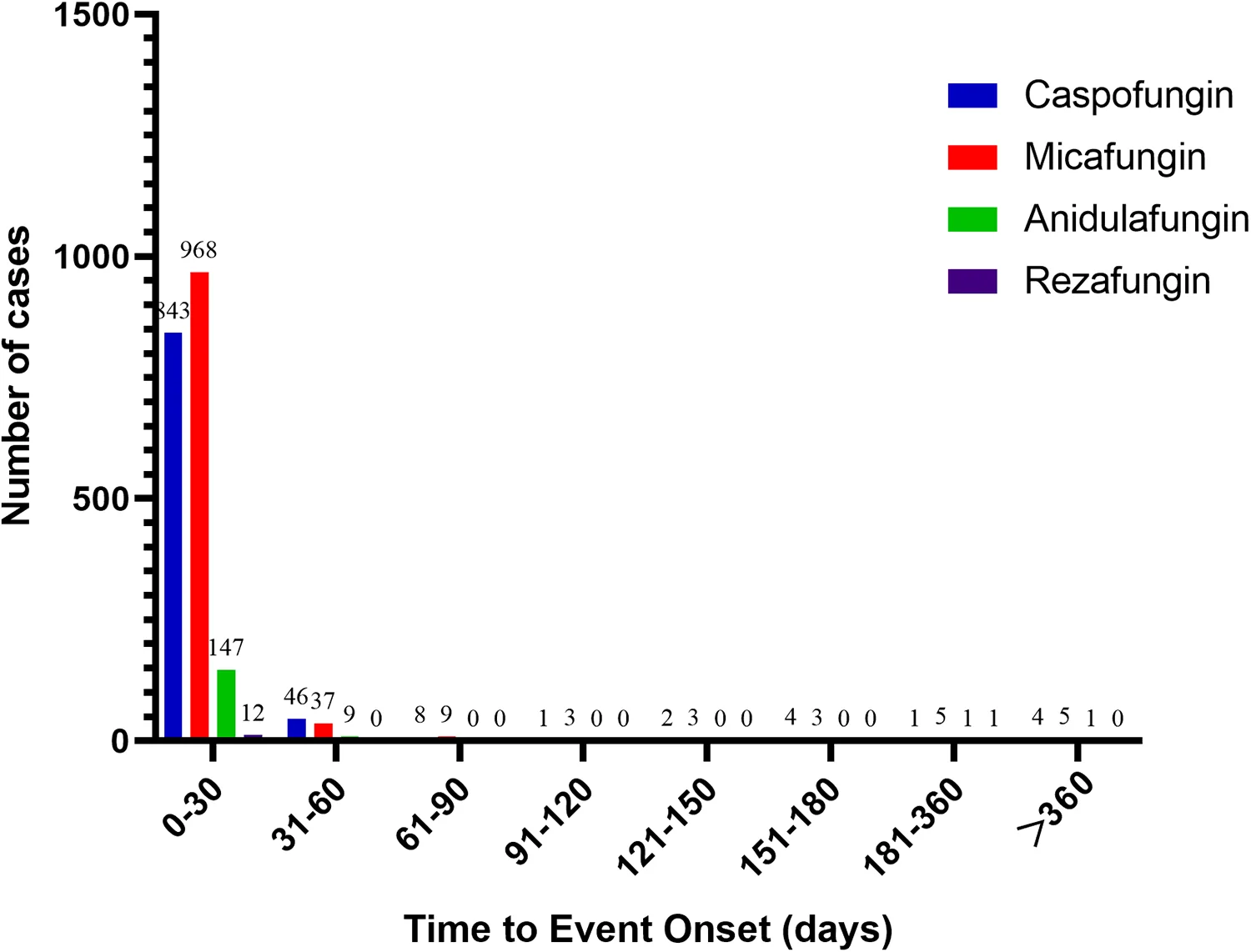
Time-to-onset distribution of adverse events for four echinocandins. The *x*-axis shows time-to-onset intervals (0–30, 31–60, 61–90, 91–120, 121–150, 151–180, 181–360, >360 days), and the *y*-axis displays the number of reports with documented dates. Stacked bars allow visual comparison across drugs. Reports with missing dates (*n* = 3,185) were excluded from this analysis.

### 3.4 Primary signal detection of adverse events

Disproportionality analysis was performed using four statistical methods: reporting odds ratio (ROR), proportional reporting ratio (PRR), information component (IC025), and empirical Bayesian geometric mean (EBGM05). An AE was considered a positive signal only if all four methods yielded positive results. Table 2 presents the detailed signal detection results for the top 20 PTs for each echinocandin.

**Table 2:** The top 20 adverse reactions of four echinocandins at the preferred term (PT) level.

PT	*N*	ROR(95% CI)	PRR(*χ*2)	IC(IC025)	EBGM(EBGM05)	Positive signal
Caspofungin						
Drug ineffective	548	4.61 (4.22–5.03)	4.28 (1407.64)	2.10 (1.96)	4.28 (3.92)	Y
Death	164	1.99 (1.70–2.32)	1.96 (78.65)	0.97 (0.74)	1.96 (1.68)	N
Product use in unapproved indication	135	5.94 (5.01–7.05)	5.83 (542.26)	2.54 (2.24)	5.83 (4.91)	Y
Multiple organ dysfunction syndrome	111	25.68 (21.28–30.99)	25.23 (2578.17)	4.65 (4.10)	25.17 (20.85)	Y
Septic shock	85	20.60 (16.63–25.52)	20.32 (1559.56)	4.34 (3.74)	20.28 (16.37)	Y
Off label use	78	0.94 (0.75–1.18)	0.94 (0.29)	−0.09(−0.41)	0.94 (0.75)	N
Sepsis	76	7.04 (5.61–8.82)	6.96 (388.34)	2.80 (2.36)	6.96 (5.55)	Y
Pneumonia	75	2.27 (1.81–2.85)	2.25 (52.42)	1.17 (0.81)	2.25 (1.79)	N
Aspergillus infection	66	89.57 (70.20–114.29)	88.61 (5666.19)	6.46 (4.90)	87.82 (68.83)	Y
Pyrexia	65	1.93 (1.51–2.47)	1.92 (28.93)	0.94 (0.57)	1.92 (1.51)	N
Pathogen resistance	60	68.83 (53.33–88.83)	68.16 (3943.30)	6.08 (4.64)	67.69 (52.45)	Y
Respiratory failure	60	8.41 (6.52–10.85)	8.34 (387.53)	3.06 (2.52)	8.33 (6.46)	Y
Bronchopulmonary aspergillosis	58	77.86 (60.06–100.94)	77.13 (4324.40)	6.26 (4.69)	76.53 (59.03)	Y
Fungal infection	56	17.36 (13.34–22.60)	17.21 (854.11)	4.10 (3.36)	17.18 (13.21)	Y
Drug resistance	55	22.32 (17.11–29.12)	22.13 (1107.48)	4.46 (3.62)	22.08 (16.93)	Y
Blood alkaline phosphatase increased	47	19.10 (14.33–25.46)	18.96 (798.50)	4.24 (3.37)	18.93 (14.20)	Y
Cholestasis	46	24.97 (18.67–33.38)	24.78 (1047.62)	4.63 (3.62)	24.72 (18.49)	Y
Renal failure	46	3.49 (2.61–4.66)	3.47 (81.01)	1.79 (1.30)	3.47 (2.60)	Y
Drug reaction with eosinophilia and systemic symptoms	46	16.62 (12.43–22.22)	16.50 (669.00)	4.04 (3.21)	16.47 (12.32)	Y
Alanine aminotransferase increased	45	7.51 (5.60–10.07)	7.46 (251.73)	2.90 (2.28)	7.45 (5.56)	Y
Micafungin						
Off label use	543	7.20 (6.59–7.87)	6.64 (2635.80)	2.73 (2.59)	6.64 (6.08)	Y
Drug ineffective	220	1.77 (1.55–2.02)	1.74 (70.91)	0.80 (0.60)	1.74 (1.52)	N
Death	204	2.53 (2.20–2.91)	2.48 (181.82)	1.31 (1.09)	2.47 (2.15)	Y
Multiple organ dysfunction syndrome	94	21.97 (17.92–26.94)	21.64 (1847.77)	4.43 (3.85)	21.59 (17.61)	Y
Product use issue	91	5.24 (4.26–6.45)	5.18 (307.68)	2.37 (2.00)	5.18 (4.21)	Y
Pyrexia	73	2.20 (1.75–2.77)	2.19 (47.33)	1.13 (0.77)	2.19 (1.74)	N
Septic shock	65	15.90 (12.45–20.31)	15.74 (896.39)	3.97 (3.33)	15.72 (12.31)	Y
Sepsis	57	5.33 (4.11–6.92)	5.29 (198.47)	2.40 (1.92)	5.29 (4.07)	Y
Bronchopulmonary aspergillosis	53	71.99 (54.88–94.43)	71.36 (3650.86)	6.15 (4.55)	70.85 (54.01)	Y
Pneumonia	53	1.62 (1.23–2.12)	1.61 (12.37)	0.69 (0.28)	1.61 (1.23)	N
Product use in unapproved indication	53	2.33 (1.78–3.05)	2.32 (39.91)	1.21 (0.78)	2.32 (1.77)	N
Aspartate aminotransferase increased	52	10.17 (7.74–13.37)	10.09 (425.93)	3.33 (2.71)	10.08 (7.67)	Y
Condition aggravated	52	1.83 (1.39–2.40)	1.82 (19.32)	0.86 (0.44)	1.82 (1.39)	N
Infection	51	3.75 (2.85–4.95)	3.73 (102.13)	1.90 (1.42)	3.73 (2.83)	Y
Respiratory failure	50	7.09 (5.37–9.36)	7.04 (259.13)	2.81 (2.25)	7.03 (5.32)	Y
Rash	49	1.12 (0.85–1.49)	1.12 (0.65)	0.17 (−0.25)	1.12 (0.85)	N
Alanine aminotransferase increased	48	8.12 (6.11–10.79)	8.06 (296.95)	3.01 (2.40)	8.06 (6.06)	Y
Candida infection	47	23.93 (17.95–31.90)	23.75 (1022.22)	4.57 (3.59)	23.70 (17.78)	Y
Hepatic function abnormal	43	12.29 (9.10–16.59)	12.20 (442.03)	3.61 (2.84)	12.19 (9.03)	Y
Renal failure	41	3.15 (2.32–4.28)	3.13 (59.66)	1.65 (1.13)	3.13 (2.30)	Y
Anidulafungin						
Death	42	2.90 (2.13–3.94)	2.82 (50.16)	1.50 (0.99)	2.82 (2.07)	Y
Drug ineffective	33	1.46 (1.03–2.07)	1.45 (4.66)	0.53 (0.01)	1.45 (1.02)	N
Off label use	32	2.20 (1.55–3.13)	2.17 (20.40)	1.12 (0.55)	2.17 (1.52)	N
Dyspnoea	29	3.00 (2.08–4.34)	2.95 (37.72)	1.56 (0.93)	2.95 (2.04)	Y
Multiple organ dysfunction syndrome	22	28.58 (18.74–43.60)	28.02 (573.38)	4.81 (3.08)	28.01 (18.36)	Y
Septic shock	19	25.91 (16.46–40.78)	25.47 (446.76)	4.67 (2.87)	25.46 (16.17)	Y
Rash	15	1.92 (1.15–3.19)	1.90 (6.47)	0.93 (0.12)	1.90 (1.14)	N
Respiratory failure	13	10.25 (5.93–17.71)	10.14 (107.16)	3.34 (1.84)	10.13 (5.86)	N
Thrombocytopenia	13	6.80 (3.94–11.75)	6.73 (63.55)	2.75 (1.48)	6.73 (3.90)	Y
Tachycardia	12	7.83 (4.43–13.83)	7.75 (70.69)	2.95 (1.55)	7.75 (4.39)	Y
Erythema	11	3.02 (1.67–5.47)	3.00 (14.69)	1.58 (0.52)	3.00 (1.65)	N
Hypotension	11	3.18 (1.76–5.76)	3.16 (16.26)	1.66 (0.58)	3.16 (1.74)	N
Pyrexia	11	1.84 (1.01–3.32)	1.83 (4.14)	0.87(−0.07)	1.83 (1.01)	N
Sepsis	11	5.70 (3.15–10.33)	5.65 (42.21)	2.50 (1.19)	5.65 (3.12)	Y
Seizure	10	3.34 (1.79–6.23)	3.32 (16.29)	1.73 (0.58)	3.32 (1.78)	N
Acute kidney injury	10	2.95 (1.58–5.49)	2.93 (12.73)	1.55 (0.44)	2.93 (1.57)	N
Hepatic failure	9	17.21 (8.93–33.18)	17.08 (136.27)	4.09 (1.79)	17.07 (8.86)	Y
Pneumonia	9	1.52 (0.79–2.93)	1.52 (1.59)	0.60(−0.39)	1.52 (0.79)	N
Drug ineffective for unapproved indication	9	9.26 (4.81–17.85)	9.19 (65.76)	3.20 (1.42)	9.19 (4.77)	Y
Candida infection	9	25.36 (13.16–48.88)	25.16 (208.76)	4.65 (1.96)	25.15 (13.05)	Y
Rezafungin						
Off label use	44	20.35 (14.56,28.44)	16.09 (631.46)	4.01 (3.11)	16.09 (11.52)	Y
No adverse event	12	22.20 (12.38,39.78)	20.92 (228.30)	4.39 (2.22)	20.92 (11.67)	Y
Product prescribing issue	12	216.73 (120.89,388.55)	203.79 (2420.56)	7.67 (2.79)	203.65 (113.59)	Y
Death	6	2.21 (0.98,4.99)	2.18 (3.88)	1.12(−0.22)	2.18 (0.97)	N
Hypokalaemia	5	34.71 (14.29,84.33)	33.87 (159.58)	5.08 (1.19)	33.86 (13.94)	Y
Drug ineffective for unapproved indication	4	22.49 (8.36,60.52)	22.06 (80.49)	4.46 (0.77)	22.06 (8.20)	Y
Cerebrovascular accident	3	5.49 (1.76,17.18)	5.43 (10.86)	2.44 (−0.09)	5.43 (1.73)	N
Dyspnoea	3	1.66 (0.53,5.18)	1.65 (0.77)	0.72 (−0.96)	1.65 (0.53)	N
Product use issue	3	5.17 (1.65,16.17)	5.11 (9.94)	2.35 (−0.13)	5.11 (1.63)	N
Product dose omission issue	3	1.86 (0.59,5.81)	1.85 (1.17)	0.88 (−0.85)	1.85 (0.59)	N
Anaphylactic shock	2	24.71 (6.14,99.49)	24.47 (45.04)	4.61 (−0.21)	24.47 (6.08)	N
Anxiety	2	2.17 (0.54,8.76)	2.16 (1.26)	1.11 (−1.04)	2.16 (0.54)	N
Back pain	2	2.68 (0.66,10.78)	2.66 (2.08)	1.41 (−0.91)	2.66 (0.66)	N
Chills	2	5.30 (1.32,21.36)	5.26 (6.92)	2.40 (−0.56)	5.26 (1.31)	N
Neutropenia	2	4.64 (1.15,18.70)	4.61 (5.66)	2.20 (−0.62)	4.61 (1.14)	N
Pneumonia	2	1.83 (0.45,7.36)	1.82 (0.74)	0.86 (−1.17)	1.82 (0.45)	N
Sepsis	2	5.60 (1.39,22.53)	5.55 (7.47)	2.47 (−0.54)	5.55 (1.38)	N
Haemodynamic instability	2	85.58 (21.25,344.65)	84.73 (165.46)	6.40 (−0.13)	84.71 (21.03)	N
Acute kidney injury	2	3.18 (0.79,12.82)	3.16 (2.96)	1.66 (−0.80)	3.16 (0.79)	N
Therapy partial responder	2	44.84 (11.13,180.55)	44.40 (84.85)	5.47 (−0.16)	44.39 (11.02)	N

For caspofungin (2,408 reports), 16 out of the top 20 PTs were positive signals; only “Death”, “Pyrexia”, “Pneumonia”, and “Off label use” did not reach the threshold. The most frequently reported PT was “Drug ineffective” (548 cases, ROR = 4.61, IC025 = 1.96). Infectious and resistance-related signals were remarkably strong: “Aspergillus infection” (66 cases, ROR = 89.57, IC025 = 4.90), “Bronchopulmonary aspergillosis” (58 cases, ROR = 77.86, IC025 = 4.69), “Pathogen resistance” (60 cases, ROR = 68.83, IC025 = 4.64), “Fungal infection” (56 cases, ROR = 17.36, IC025 = 3.36), and “Drug resistance” (55 cases, ROR = 22.32, IC025 = 3.62) all demonstrated strong positive signals. Serious systemic events also yielded strong positive signals: “Multiple organ dysfunction syndrome” (MODS) (111 cases, ROR = 25.68, IC025 = 4.10), “Septic shock” (85 cases, ROR = 20.60, IC025 = 3.74), “Sepsis” (76 cases, ROR = 7.04, IC025 = 2.36), and “Respiratory failure” (60 cases, ROR = 8.41, IC025 = 2.52). Hepatobiliary and renal abnormalities were positive signals: “Blood alkaline phosphatase increased” (47 cases, ROR = 19.10, IC025 = 3.37), “Cholestasis” (46 cases, ROR = 24.97, IC025 = 3.62), “Renal failure” (46 cases, ROR = 3.49, IC025 = 1.30), and “Alanine aminotransferase increased” (45 cases, ROR = 7.51, IC025 = 2.28). Furthermore, “Drug reaction with eosinophilia and systemic symptoms” (DRESS) was a positive signal (46 cases, ROR = 16.62, IC025 = 3.21), indicating a risk of severe hypersensitivity. A sensitivity analysis restricted to reports where caspofungin was the sole suspected drug yielded 31 DRESS cases with ROR = 14.89 (IC025 = 3.05), confirming the signal remained positive (Supplementary Table S4).

For micafungin (2,375 reports), 14 out of the top 20 PTs were positive signals. Positive signals included: “Off label use” (543 cases, ROR = 7.20, IC025 = 2.59), “Death” (204 cases, ROR = 2.53, IC025 = 1.09), “Multiple organ dysfunction syndrome” (94 cases, ROR = 21.97, IC025 = 3.85), “Product use issue” (91 cases, ROR = 5.24, IC025 = 2.00), “Septic shock” (65 cases, ROR = 15.90, IC025 = 3.33), “Sepsis” (57 cases, ROR = 5.33, IC025 = 1.92), “Bronchopulmonary aspergillosis” (53 cases, ROR = 71.99, IC025 = 4.55), “Aspartate aminotransferase increased” (52 cases, ROR = 10.17, IC025 = 2.71), “Infection” (51 cases, ROR = 3.75, IC025 = 1.42), “Respiratory failure” (50 cases, ROR = 7.09, IC025 = 2.25), “Alanine aminotransferase increased” (48 cases, ROR = 8.12, IC025 = 2.40), “Candida infection” (47 cases, ROR = 23.93, IC025 = 3.59), “Hepatic function abnormal” (43 cases, ROR = 12.29, IC025 = 2.84), and “Renal failure” (41 cases, ROR = 3.15, IC025 = 1.13). Of note, “Drug ineffective” (220 cases, ROR = 1.77, IC025 = 0.60) did not reach the positive threshold, differing from caspofungin. The signal strength for “Bronchopulmonary aspergillosis” (ROR = 71.99) was comparable to that of caspofungin, suggesting similar challenges in treating aspergillus infections.

For anidulafungin (416 reports), 10 out of the top 20 PTs were positive signals. Positive signals included: “Death” (42 cases, ROR = 2.90, IC025 = 0.99), “Dyspnoea” (29 cases, ROR = 3.00, IC025 = 0.93), “Multiple organ dysfunction syndrome” (22 cases, ROR = 28.58, IC025 = 3.08), “Septic shock” (19 cases, ROR = 25.91, IC025 = 2.87), “Thrombocytopenia” (13 cases, ROR =  6.80, IC025 = 1.48), “Tachycardia” (12 cases, ROR = 7.83, IC025 = 1.55), “Sepsis” (11 cases, ROR =  5.70, IC025 = 1.19), “Hepatic failure” (9 cases, ROR = 17.21, IC025 = 1.79), “Drug ineffective for unapproved indication” (9 cases, ROR = 9.26, IC025 = 1.42), and “Candida infection” (9 cases, ROR =  25.36, IC025 = 1.96). “Drug ineffective” (33 cases, ROR = 1.46, IC025 = 0.01), “Off label use” (32 cases, ROR = 2.20, IC025 = 0.55), and “Respiratory failure” (13 cases, ROR = 10.25, IC025 = 1.84, negative due to other criteria) did not reach the positive threshold. Unlike caspofungin and micafungin, anidulafungin showed no signal for aspergillus or drug resistance.

For rezafungin (99 reports), most PTs had fewer than 10 cases. Among the top 20 PTs, only 5 were positive signals, which should be interpreted with caution. Positive signals included: “Off label use” (44 cases, ROR = 20.35, IC025 = 3.11), “No adverse event” (12 cases, ROR = 22.20, IC025 = 2.22), “Product prescribing issue” (12 cases, ROR = 216.73, IC025 = 2.79), “Hypokalaemia” (5 cases, ROR = 34.71, IC025 = 1.19), and “Drug ineffective for unapproved indication” (4 cases, ROR = 22.49, IC025 = 0.77). “Death” (6 cases, ROR = 2.21, IC025 = −0.22), “Cerebrovascular accident” (3 cases), and “Dyspnoea” (3 cases) were all negative. Signals for rezafungin were predominantly related to medication practice issues (off-label use, prescribing errors) and laboratory abnormality (hypokalaemia), with no signals for infection, resistance, or DRESS.

The similarities and differences in AE signals among the four echinocandins are largely driven by reporting volume. Caspofungin and micafungin, with wider use and more reports, exhibited the richest positive signal profiles. Anidulafungin and rezafungin, with fewer reports, had limited signal detectability. For domains with sufficient reporting across drugs (e.g., serious systemic events, hepatic abnormalities), the four agents showed similar trends. Resistance-related signals were detected only for caspofungin and micafungin, and the DRESS signal was unique to caspofungin. These differences merit further validation in larger, more balanced datasets.

Note: “Y” indicates that all four disproportionality methods (ROR, PRR, IC025, and EBGM05) yielded positive results, and the adverse event was considered a positive signal. “N” indicates that at least one of the four methods did not reach the positive threshold, and the event was not considered a positive signal.

### 3.5 Distribution of adverse events by system organ class

As illustrated in Figure 3, AE reports for echinocandins were classified by MedDRA SOC and sorted by the proportion of reports. The highest proportion was observed for Infections and infestations, consistent with the strong positive signals for Aspergillus infection, fungal infection, and sepsis at the PT level. General disorders and administration site conditions ranked second, encompassing events such as drug ineffective and multiple organ dysfunction. Hepatobiliary disorders and Investigations ranked third and fourth, corresponding to elevated transaminases and cholestasis. Respiratory, thoracic and mediastinal disorders and Renal and urinary disorders also contributed notable proportions. SOCs such as Pregnancy, puerperium and perinatal conditions, Social circumstances, and Product issues had extremely low reporting proportions. The SOC-level distribution corroborates our PT-level signal detection, indicating that AEs associated with echinocandins are predominantly concentrated in infectious complications, systemic disorders, hepatobiliary injury, and renal abnormalities.

**Figure 3:**
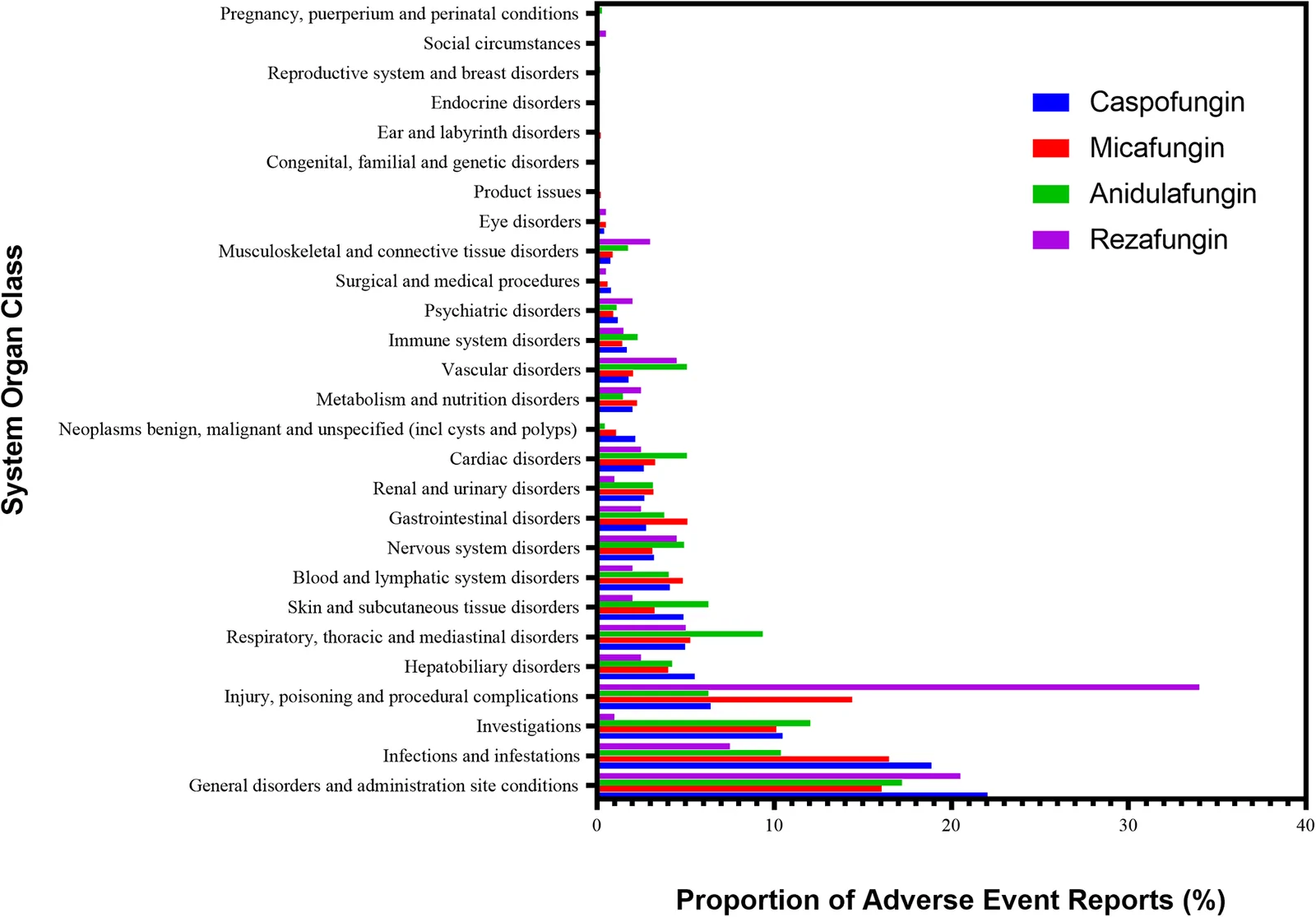
Distribution of echinocandin-related AE reports by system organ class (SOC). The *y*-axis lists SOC categories, and the *x*-axis represents the percentage of total reports for each drug. Bar length indicates the relative proportion of reports for each SOC. Reports with multiple PTs were counted separately for each SOC.

## 4 Discussion

This large-scale pharmacovigilance study systematically analyzed echinocandin-associated AEs using FAERS data spanning over 21 years. While our findings confirm several known safety signals (hepatobiliary toxicity, serious systemic events), two distinctive discoveries warrant particular attention: (1) the robust and unique association between caspofungin and DRESS syndrome, which has not been previously quantified in large pharmacovigilance studies; and (2) the striking strength of resistance-related signals for caspofungin and micafungin, which carry important implications for antifungal stewardship. These two novel findings distinguish our study from previous reports and offer actionable insights for clinicians.

The most notable and novel finding of this study is the positive signal for DRESS uniquely associated with caspofungin (ROR = 16.62, IC025 = 3.21). DRESS is a rare but potentially life-threatening hypersensitivity syndrome with a mortality rate of up to 10% (17). It typically presents with rash, fever, lymphadenopathy, eosinophilia, and visceral involvement (hepatitis, nephritis, pneumonitis, myocarditis) (18). However, no previous pharmacovigilance study has quantified this risk at a population level. Our finding that DRESS was a positive signal for caspofungin, but did not reach the top 20 threshold for micafungin, anidulafungin, or rezafungin, suggests a potential drug-specific association. However, this absence from the top 20 PTs should not be interpreted as evidence of safety for the other echinocandins. Differences in approval dates, global usage, reporting volumes, and reporter characteristics may substantially affect signal detection. We therefore caution against overinterpreting the absence of DRESS signals for these agents as confirming their absence of risk.

Several lines of evidence support the biological plausibility of this finding. Mechanistic studies have suggested that caspofungin may activate macrophages via Mas-related G protein-coupled receptor X2 (MRGPRX2), triggering mast cell activation and anaphylactoid reactions (19). This pathway is distinct from classic IgE-mediated hypersensitivity and may explain the delayed onset and systemic nature of DRESS. Additionally, a *post-hoc* pooled analysis of the phase 2 STRIVE trial and the phase 3 ReSTORE trial identified one case of anaphylactic shock in a patient receiving caspofungin (20), further corroborating the potential for severe hypersensitivity reactions with this agent. We acknowledge that other medications (e.g., antibiotics, antiepileptic drugs, allopurinol) may contribute to DRESS and cannot be fully excluded in FAERS data. To address this, we performed a sensitivity analysis restricted to cases where caspofungin was the sole suspected drug. The DRESS signal remained positive (ROR = 14.89, IC025 = 3.05) based on 31 such reports, supporting the robustness of the association and reducing the likelihood that the signal was driven solely by concomitant medications. The clinical implications of this finding are substantial. First, clinicians should maintain a high index of suspicion for DRESS when patients receiving caspofungin develop persistent fever, skin eruption, eosinophilia, or unexplained visceral organ dysfunction. Second, early recognition and prompt withdrawal of the offending drug are critical, as delayed diagnosis is associated with worse outcomes. Third, given that DRESS was not detected for other echinocandins, clinicians may consider switching to micafungin or anidulafungin when DRESS is suspected, although cross-reactivity cannot be entirely excluded. Fourth, we recommend baseline eosinophil counts and weekly monitoring during caspofungin therapy, particularly in patients with known drug allergies or a history of hypersensitivity reactions.

The second major finding of this study is the extraordinary strength of resistance-related signals for caspofungin and, to a lesser extent, micafungin. Caspofungin generated positive signals for “pathogen resistance” (ROR = 68.83, IC025 = 4.64) and “drug resistance” (ROR = 22.32, IC025 = 3.62), while micafungin showed a strong signal for “bronchopulmonary aspergillosis” (ROR = 71.99, IC025 = 4.55) and “Candida infection” (ROR = 23.93, IC025 = 3.59). These ROR values far exceed typical thresholds for confounding by indication, suggesting that clinical failure and suspected resistance are being increasingly recognized in real-world practice. While PTs such as “Aspergillus infection,” “bronchopulmonary aspergillosis,” and “fungal infection” partly reflect the underlying indications for echinocandin therapy, the extremely high ROR values (up to 89.57 for caspofungin and 71.99 for micafungin) suggest that these signals may also capture clinical treatment failure or persistent infection in real-world practice. However, these signals do not confirm microbiologically confirmed resistance (e.g., FKS mutations); they reflect reporting of suspected resistance or treatment failure. Confirmation requires antifungal susceptibility testing and genetic analysis.

Echinocandin resistance, although still relatively rare, is an emerging threat, particularly in *Candida glabrata* (*Nakaseomyces glabratus*), where FKS hotspot mutations confer cross-resistance to all echinocandins (13, 21). A 12-year longitudinal study from China collected 11,679 Candida isolates and observed cross-resistance and multidrug resistance to azoles and echinocandins across all major Candida species (22). Our pharmacovigilance data complement these microbiological findings, demonstrating that resistance is not just a laboratory phenomenon but a clinically observable reality captured in spontaneous reporting systems. Notably, resistance-related signals were absent for anidulafungin and rezafungin. This absence, however, should not be interpreted as evidence of lower resistance risk. Anidulafungin had only 416 reports, and rezafungin only 99, which precludes reliable signal detection for rare events. Importantly, *in vitro* studies have shown that rezafungin selects for FKS mutants at a frequency similar to that of anidulafungin and caspofungin (23). Thus, absence of a signal does not prove absence of risk, a critical caveat that clinicians and researchers must bear in mind. The implications for clinical practice are clear. First, antifungal susceptibility testing should be performed when clinical response is suboptimal after 5 to 7 days of echinocandin therapy. Second, empirical switching between echinocandins may not overcome resistance caused by FKS mutations, as these confer pan-echinocandin cross-resistance. Third, antifungal stewardship programs should monitor local resistance patterns and implement surveillance systems to detect emerging resistance early. Our findings provide real-world evidence supporting these stewardship efforts.

In addition to the two core findings, our study confirmed several known safety signals consistent with previous literature. Hepatobiliary AEs, including cholestasis and alkaline phosphatase elevation for caspofungin, and elevated transaminases, hepatic function abnormalities, and hepatic failure for micafungin and anidulafungin respectively, were robust positive signals, consistent with product labels and meta-analyses (24). Mechanistically, NTCP/Ntcp and BSEP/Bsep are primarily responsible for micafungin's hepatobiliary disposition, and disruption of bile acid handling may underlie micafungin-associated hepatotoxicity (25). PI3 K/Akt and HIF-1 pathways have been implicated in caspofungin-induced liver injury (11). Clinicians should monitor liver function weekly during prolonged courses (≥14 days).

Serious systemic events, including MODS, septic shock, sepsis, and respiratory failure, were positive across all four drugs, reflecting the severe underlying conditions of patients receiving echinocandins rather than drug-specific toxicity. Nevertheless, clinicians should be aware that rapid clinical deterioration may warrant consideration of drug discontinuation, especially when other causes have been excluded.

Based on our two core findings, we propose the following prioritized clinical recommendations. For caspofungin, clinicians should monitor eosinophil counts at baseline and weekly during therapy; if fever, rash, eosinophilia, or visceral dysfunction develops, DRESS should be suspected, the drug should be discontinued immediately, and switching to another echinocandin such as micafungin or anidulafungin should be considered. For all echinocandins, antifungal susceptibility testing should be performed if clinical response is poor after 5 to 7 days of therapy, and clinicians should not assume that switching between echinocandins will overcome resistance, as FKS mutations confer cross-resistance. For resistance surveillance, local antifungal stewardship programs should be implemented to monitor resistance patterns and guide empirical therapy.

Several limitations warrant acknowledgment. FAERS is a spontaneous reporting system subject to under-reporting, selective reporting, and confounding by indication. The absence of denominator data precludes incidence calculation. US reports dominated, limiting generalizability. The time-to-onset analysis was compromised by data extraction artifacts. Rezafungin data are limited due to recent approval. Despite these limitations, the two core signals, DRESS for caspofungin and resistance for caspofungin/micafungin, are robust, biologically plausible, and clinically actionable.

Future research should focus on prospective registries to validate the DRESS-caspofungin association, pharmacogenomic studies to identify HLA or MRGPRX2 polymorphisms conferring susceptibility, and surveillance networks to monitor echinocandin resistance trends globally.

## 5 Conclusion

This study identifies two distinctive and clinically significant signals for echinocandins: DRESS as a unique safety signal for caspofungin, quantified for the first time in a large pharmacovigilance database; and exceptionally strong resistance-related signals for caspofungin and micafungin, underscoring the urgent need for antifungal stewardship. These findings differentiate our study from previous reports and provide actionable guidance for clinicians. Further high-quality studies are warranted to confirm causality and explore underlying mechanisms.

## Data Availability

The original contributions presented in the study are included in the article/Supplementary Material, further inquiries can be directed to the corresponding author.
